# Approaches towards averting a potential structural shortage of general practitioners: results of a quantitative survey on attitudes, experiences, and ideas from general practitioners in the Federal Republic of Germany

**DOI:** 10.1186/s12875-025-02840-y

**Published:** 2025-04-24

**Authors:** Julian Wangler, Stefan Claus, Michael Jansky

**Affiliations:** Centre for General Medicine and Geriatrics, University Medical Center of the Johannes Gutenberg, University Mainz, Am Pulverturm 13, 55131 Mainz, Germany

**Keywords:** GP shortage, Rural physician, Country practice, Primary care, Established care

## Abstract

**Background:**

Securing primary care poses a complex set of challenges for policymakers in national healthcare. The risk of a shortage in general practitioners raises the question as to which approach would make an effective contribution towards averting an impending healthcare shortage. There has been a lack of studies shedding light on how general practitioners pview various approaches towards securing long-term primary care, and which measures they support from their own professional experience. The aim of the study was to ascertain the opinions, attitudes, and experiences of general practitioners in securing primary care across the country. We ran a survey to ask GPs about strategies that they saw as promising or particularly pressing, how they viewed the current situation regarding the shortage of general practitioners, and what they saw as causes for any decline they had already seen in primary care.

**Methods:**

Our survey conducted online addressed a total of 5,164 general practitioners interviewed between August 2023 and April 2024 (40% response rate). Apart from descriptive analysis, we used Student’s t-test for independent samples to determine significant differences between two groups. We also performed a factor analysis (Varimax rotation).

**Results:**

Our respondents displayed a somewhat negative view of primary care development in Germany. Many general practitioners expressed concerns as to whether primary care would remain secure in the long term. Despite partial progress, respondents saw a great need for countermeasures in the coming years. Nearly half of the respondents at 44% saw a noticeable decrease in general practices in their local area. Physicians in small towns and rural communities were more than twice as likely to experience the healthcare shortage due to a decline in general practitioners compared to their colleagues in medium-sized and large cities (42% versus 19%, *p*<0.001). More than half at 55% reported declining attractiveness in primary care for young physicians, which they attributed to three problem areas: 1) Position of primary care within the healthcare system, 2) Requirements for foundational and continuing medical education, 3) Working conditions. Respondents especially advocated the following approaches toward securing primary care: Establishing a primary care system (88%), more intense promotion of interest in primary care with points of contact in foundational and continuing medical education, especially in accompanying longitudinal programmes (78%), reinforcing multi-professional outpatient care centres (62%), restructuring curricula (54%) and enrolment criteria for medical study courses (51%), and reforming general medical training (55%).

**Conclusions:**

General practitioners have their own proposals and preferences to add to the existing assessments and expert opinions. General practitioners should be involved more consistently than before in planning, implementing, and evaluating measures towards stabilising primary care. Various modes of participation and collaboration exist to this end.

**Supplementary Information:**

The online version contains supplementary material available at 10.1186/s12875-025-02840-y.

## Background

Health policy in Germany has shown increasing concern about the potential scenario of an impending shortage of primary care. Intensified exchanges have also been taking place in primary care and the broader scientific community on the likelihood, risk, and options involved in preventing this development [1]. The socio-demographic situation shows every third general practitioner to be at least 60 years old [2, 3]. Germany is not the only European nation facing this issue, but ageing amongst general practitioners is especially pronounced in Germany due to the more rapid demographic change in this country [3]. Statistics from the National Association of Statutory Health Insurance Physicians (KBV) have shown around four thousand general practitioner vacancies in 2021 with an average of 60% of vacancies for general practitioners within the statutory health insurance system remaining unfilled in recent years [2]. Other European countries have also been showing signs of structural issues in maintaining general practitioners’ practices or recruiting new physicians to practise there. These signs take different forms due to the varying nature of health systems, but there has been a fundamental trend towards precariousness in the healthcare situation in highly rural, structurally weak areas almost everywhere [4–7]. A worsening shortage of general practitioners has been widely reported in the public and the mass media, especially involving succession failures in general practices [8–17].

Around 1,700 general practitioners retire every year according to calculations, compared to around 1,300 to 1,400 specialists [18, 19]. The number of specialists may have been increasing recently, yet the need to make up for the shortage and maintain existing capacity has remained unmet for a lengthy period. General practitioners joining the profession have been showing a greater preference for part-time work and more flexible working models as well as permanent employment, worsening the situation [20–23]. In contrast, the classical solo practice has come under pressure with young physicians showing a widespread aversion to risk regarding entrepreneurial independence and practice management [3, 18, 19]. These developments in combination could lead to a deficit equivalent to up to 23,000 full-time primary care physicians by 2030, with rural and structurally weak regions likely to be especially severely impacted [24, 25]. A 2021 study by the Robert Bosch Foundation predicts a shortage in forty percent of all German districts by 2035 in the worst case [19, 26]. Increasing patient numbers concentrated into fewer remaining practices will increase physical and psychological stress while also eroding job satisfaction amongst general practitioners [6, 12, 16, 26].

These findings and projections have led to ongoing discussion on suitable measures to counteract a potentially severe and preventable decline in general practitioners as effectively as possible [27]. One tentative focus has been placed on structural reinforcement of primary care by widespread creation of multi-professional outpatient centres to make up for the decline in traditional individual practices while also fostering interdisciplinary involvement in primary care [28]. Various stakeholders have also been calling for a primary care physician system aiming at a substantial enhancement of the role of general practitioners, thus increasing the attractiveness of a career in general practice [29, 30]. Even so, there is no consensus amongst health policy and scientific circles at the moment as to whether a primary care physician system would actually bring about a noticeable improvement the position of primary care in the German healthcare system [31, 32]. Another approach continuously pursued for years involves creating new delegation models within primary care towards relieving general practitioners while also increasing healthcare efficiency [23, 31].

Several strategies are being developed or implemented towards encouraging general practitioners to set up practices, especially in rural areas. Examples include stronger demand planning and distribution with more impact at regional level [33], but also a more effective incentive structure using establishment premiums, investment cost subsidies, and bursaries. Suggestions also include improvements in structuring in continuing medical education at district and municipality level as well as by associations of statutory health insurance physicians, primary care institutes, and statutory health insurance organisations [34]. Increasing the share of general practitioners in further education, setting quotas for access to specialist training, and providing more access to general practice from other disciplines under certain conditions have also been proposed as measures towards expanding the pool of general practitioners [30, 35].

Far-reaching proposals have been raised towards adapting foundational and continuing medical education in addition to rural GP quotas at federal state level. These proposals focus on redesigned curricula in human medicine courses while also adjusting enrolment criteria to encourage general practice as a career opportunity [36]. Finally, recommendations have been given to change the type and duration of specialist training with a more concerted focus on primary care [37].

As studies and expert opinions emphasise, no single priority measure will be sufficient given the complexity of challenges facing future developments in primary care. Rather, a coordinated, varied, and in some aspects regionally based package of measures would be necessary to counteract the substantial shortfall in primary care [30].

Considering the large numbers of protagonists and policymakers in health policy and health services research commenting on the topic, the lack of demand for original perspectives from primary care has been striking. This has led to a lack of documentation on how general practitioners view these various approaches towards securing long-term primary care based on their own professional experience [6, 23, 30]. Including the views and assessments of general practitioners in planning, implementing, and fine-tuning these measures would seem necessary towards achieving sustainable solutions compatible with primary care.

### Research interest

The aim of the study was to obtain the opinions, attitudes, and experiences of general practitioners towards securing long-term general medical care. We focused on the following questions:

- How do GPs see the future of primary care?
- How secure do GPs see primary care in the future?
- Which measures would GPs consider promising or pressing in securing primary care?
- How satisfied are GPs with efforts that have been made so far?
- To what extent are differences noticeable between different subgroups, e.g. with regard to urban and rural physicians?

## Methods

### Study design and survey instrument

We conducted a full survey amongst general practitioners in four federal states in Germany from summer 2023 to spring 2024. This involved designing an online survey with a written cover letter sent in the regular mail.

A qualitative preliminary study surveying thirty-six general practitioners on the topic in 2022 [38] largely served as a basis in designing the survey used in this quantitative cross-sectional study (see Appendix 1). We also included a literature search on the topic in developing the study design (including [5, 18, 20, 23, 27, 30, 39, 40]). We especially focused on the work of van den Bussche [30], who discussed the problems of concerted measures towards securing primary care. This allowed us to draw up a list of measures to ask general practitioners about. We also adapted two questions using the 2022 MLP health report [41].

The final questionnaire contained twenty-three questions focused mainly on the following areas:

- Current situation and development of primary care in the longer term
- Personal experience of stress and observations on the shortage of physicians
- Preferred approaches or measures towards securing primary care
- Assessment of measures taken towards securing primary care as well as further optimisation approaches to be adopted

In addition to standardised questions, we included several open questions (4, 6, 9, 11, 16, 18, 20, 21) to reflect the exploratory nature of the study. The open questions touched on all of the areas mentioned and were intended as a supplement to give respondents the opportunity to verbalize their thoughts and suggestions.

We noted sociodemographic characteristics including gender, age, practice setting, type of practice, and patients seen per quarter. We performed a pretest before field use; this involved presenting the questionnaire to fifty randomly selected general practitioners among general practice lecturers in the general medicine faculty. The pretest showed answer categories to be easy to understand, well structured, and complete. To assess reliability, Cronbach's alpha was calculated; it was 0.82.

### Recruitment and participants

We sent out postal invitations to take part in an anonymised survey to all 12,671 active general practitioners in North Rhine-Westphalia (5,842), Lower Saxony (5,218), and Thuringia (1,611) between August 2023 and April 2024. We selected these particular federal states as our aim was to include densely populated territorial federal states for at least an approximation of the actual primary care situation in Germany. The second reason was that the authors had current and complete contact lists due to continuous research into primary care in these federal states. This was a one-off mailshot where potential respondents were informed of password-protected access to the online survey (no incentives), inter alia.

### Data analysis

We used SPSS 23.0 for data analysis. Student’s t-test for independent samples was used to determine significant differences between two groups. Two levels of significance were tested – mean difference at *p* < 0.05 and *p* < 0.001. This parametric method has high test power and is considered statistically robust. We satisfied the necessary conditions with the number of cases, normal distribution in groups for differentiation, and samples originating from the same population [42].

We also performed a factor analysis (Varimax rotation). Factor analysis serves to combine a larger number of variables into factors based on internal correlations. The aim of this is to reveal common underlying factors. The Varimax method that we selected is the most common method for arriving at interpretable factor solutions. We selected a 0.4/− 0.4 limit value for factor loadings [43]. The Bartlett sphericity test was performed to test for the requirements of factor analysis. This tests the hypothesis that all correlation coefficients in the population have a zero value. A significant result allows the interpretation that at least some variables correlate within the population; the null hypothesis can be rejected.

We evaluated open questions using post-coding for qualitative content analysis. This involved creating a basal category system for answers in free text to each open question [44]. STROBE was used as the reporting statement. The results as presented below include concise free-text answers.

## Results

### Sample

A total of 5,164 completed questionnaires of the 5,485 questionnaires processed were included in evaluation (40% response rate). Table 1 compares the sample obtained against the reference data from the German Association of Statutory Health Insurance Physicians (KV) on the structure of general practitioners in Germany (see Table 1). 69% of respondents answered most or all of the open questions, although the length of the text answers varied greatly.

**Table 1:** Sample comparison compared to reference statistics

	Sample (N = 5,164)	Reference statistics
Gender:	60% male 40% female	51% male 49% female1
Mean age:	52 (median: 52)	55 (median: 56) ^1^
Practice setting:	48% medium and large cities 52% rural/small town	41% medium and large cities 59% rural/small town^1^
Type of practice:	52% solo practices 36% group practices 12% polyclinics or other establishments	66% individual general practices 23% general practice group practices 11% medical care centres or other facilities^1^
Patients per quarter:	25% 500–1,500, 36% 1,501–2,000, 39% > 2000	Complete data unavailable
Academic teaching physicians:	9%	Complete data unavailable

^1^Based on the statistical information from the healthcare sector by the National Association of Statutory Health Insurance Physicians, Germany

(as of: 31 December 2022),

available at: https://gesundheitsdaten.kbv.de/

### Current situation and development of primary care in the longer term

Most respondents took rather a negative view of the current state of the healthcare system in Germany. A quarter at 25% expected overall healthcare to improve (14%) or remain the same (11%) in the coming years. In contrast, 60% assumed that there would be a moderate (41%) or substantial (19%) deterioration in the general supply situation in Germany; 14% responded that it was hard to say or didn't know. The assessment turned noticeably more negative when asked specifically about the outpatient care situation. Less than a quarter of respondents at 22% thought that outpatient healthcare would improve (10%) or remain the same (12%) in the coming years, while 68% expected a moderate (29%) or even substantial (39%) deterioration; 10% responded that it would be hard to say or didn’t know. Similarly, only 21% believed that primary care as it existed at the time of the survey was secured for the next one to two decades; more than three-quarters at 77% expected the situation to deteriorate (37%) or even lose all stability (40%). All of 20% were very (4%) or somewhat (16%) confident in contrast to 75% somewhat (38%) or very worried (37%) about future development prospects in outpatient primary care.

*“For some reason, we’ve spent far too much time blindly assuming that some of the students would automatically become general practitioners after graduation – a false assumption that is now costing us dearly.”* (Open answer from a responding GP [f])

*“We’ve been visibly in danger of running out of GPs for some time because there are not enough of them joining the system. [...] It’s been a lasting lack of action rather than a lack of awareness. They set the levers too late towards adapting the system.”* (Open answer from a responding GP [m])

Analysing the questions addressed above demonstrates that physicians in small towns and rural communities shared a substantially dimmer assessment of the future of general practice compared to physicians in large and medium-sized cities (see Table 2). There were no noticeable differences in other sociodemographic characteristics such as age or gender.

**Table 2:** Assessments on securing outpatient and primary healthcare

Questions 1, 2, 8 and 10 (N = 5,164)	Overall agreement	Urban vs. rural physicians
**Question:** *In your opinion, will healthcare in Germany improve or worsen overall in the coming years?*	60% (Somewhat worsen/substantially worsen)	53%/66%
**Question:** *In your opinion, how would you rate this for outpatient care in particular, that is, for general practitioners and specialists? Do you think these areas of healthcare will…*	68% (Somewhat worsen/substantially worsen)	54%/82%*
**Question:** *Thinking about the future and development opportunities in primary care over the next ten to twenty years, would you say you were more confident or more worried?*	75% (Somewhat worried/very worried)	62%/87%*
**Question:** *Thinking about the long term, how well would you say that primary care in Germany has been secured for the coming decades?*	77% (Somewhat insecure/not at all secure)	66%/88%*

Difference significant at *p < 0.001

Taking the entire sample into account, 35% of all respondents expected there to be an (increased) shortage of general practitioners in the next ten to twenty years, mainly in rural and structurally weak regions. Apart from that, 38% anticipated a widespread shortage of general practitioners in urban catchment areas as well (15% did not expect any major shortage). Among those respondents who perceived a shortage of general practitioners, 29% saw a future supply deficit of between 5 and 15% compared to 58% expecting a shortfall of 15% or more (13% found it hard to say or didn’t know).

A combined analysis on two open questions (9, 11) reveals that many respondents anticipated a shortfall in significant parts of Germany; this would in turn have an impact on referrals to other levels of care. In addition, a large percentage of respondents feared a vicious circle affecting the attractiveness of primary care amongst young physicians due to the increased burden on remaining general practices. To make matters worse, budget and resource restrictions imposed by the health system attracted widespread criticism in view of current health policy and the hesitant relief it has been giving.

*“I see a real danger in an extremely perilous vicious circle eventually causing disintegration in the foundations of the general practitioner profession.”* (Open answer from a responding GP [f])

More than half of the respondents at 55% reported a decrease in the attractiveness of general practice for young physicians, with the decline either strong (25%) or moderate (30%) compared to 33% expecting the profession’s attractiveness to increase strongly (6%) or moderately (27%); 11% found it hard to say or didn’t know). A large share of respondents responded to an open question with the fundamental issue concerning an image problem deterring young physicians from the profession.

*“It’s not because they won’t earn enough that young doctors aren’t joining GP practices anymore, it’s because they see the conditions as too unattractive.”* (Open answer from a responding GP [f])

Based on an evaluation of open questions 4, 6, 9 and 11, respondents mainly attributed this lack of attractiveness to three problem areas:

- Position of primary care in the German healthcare system: The respondents pointed out that division of labour between healthcare sectors was too weakly regulated and therefore ineffective. This places a disproportionate burden on general practitioners in terms of economics, time, and resources. Lack of general practitioner involvement in the interprofessional arena leads to developments such as unnecessary redundancy and potentially poorer healthcare. Young physicians are aware of the adversity involved in the role of general practitioners, dissuading them from considering primary care as a field of activity.
- Requirements for foundational and continuing medical education: Lack of preparation for outpatient and primary care in the curriculum has caused many respondents to see current losses even amongst those especially committed to primary care. This problem becomes especially apparent in self-employment and practice management. Apart from that, the value, everyday life, and motivational factors in primary care are not communicated to a sufficient degree.
- Working conditions: Respondents also criticised health policy for neglecting changing ideas on employment or work-family compatibility in the form of new employment models (such as more part-time options, flexible working hours, less desire for independence, stronger interdisciplinary links) for far too long. This has caused general practice medicine to fall behind, as it is still too focused on the classical individual practice in Germany.

Despite widespread scepticism as to future developments in primary care, a high percentage of respondents (69%) stated that they would currently recommend primary care as a profession to medical students and trainee physicians. Even considering the substantial stress factors in everyday practice, the respondents justified this response in the high level of job satisfaction, intrinsic motivation, and the belief that young physicians could provide genuine primary care and broad-based patient services in this field. A high proportion of respondents responding to open questions reported the enormous joy and fulfilment that they drew from their profession, emphasising the fundamental role of primary care. In the future, there would be *“no replacement for general practitioners as all-rounders and guides to the health system”* covering the entire spectrum of care and acting as a competent referrer.

*“The GP profession is probably more important and in demand than ever before. Even so, it’ll take fundamental decisions to meet this demand. […] This general rethinking is lacking.”* (Open answer from a responding GP [m])

### Personal experience of stress and observations on the shortage of physicians

In contrast to other surveys, our survey included a series of questions on the current situation in the respondents’ own practices and environment. A substantial percentage of the general practitioners responding saw a noticeable decline in primary care in their own practice environment. Just under half at 44% reported that their own area was highly (19%) or moderately (25%) affected by the decline in general practitioner practices, while 28% saw a slight to moderate impact (28% hardly or not at all affected). Just under a third of respondents at 31% saw the current decline in GP practices as so great that the hindrances and difficulties experienced in their own practice environment had become palpable; 28% saw a deficit in healthcare without it becoming a major issue yet. Physicians in small towns and rural communities were more than twice as likely to experience the healthcare shortage due to a decline in general practitioners compared to their colleagues in medium-sized and large cities (42% versus 19%, *p* < 0.001).

Asked about various stress factors in primary care, an overwhelming majority of respondents reported issues such as extensive, time-consuming bureaucratic obligations and perceived cost pressure, sometimes affecting their ability to provide optimal and individual healthcare (see Table 3). Physicians in rural areas especially experienced difficulties in finding new staff, direct and indirect consequences of declining primary care, and limited availability of specialists as stressful and/or limiting factors in their activities.

**Table 3:** Issues experienced in general practice

**Question:** *In your experience, how much of an impact have the following issues had on your activities as a general practitioner?* (N = 5,164; response categories *extreme* and *somewhat* taken together)	Overall agreement	Urban vs. rural physicians
Bureaucracy (such as reporting and documentation obligations)	91%	89%/92%
Cost pressure and restrictions in healthcare (such as those causing restrictions to optimal, individual patient care)	91%	91%/91%
Difficulties in finding staff (such as practice staff, physicians for employment)	78%	64%/91%*
Additional burdens from the shortage of physicians (such as your practice having to provide more patient care due to other general practices closing for lack of succession)	63%	40%/85%*
Lack of specialists available in your area for you to perform your own obligations in guiding your patients through the system properly	48%	34%/62%*

Difference significant at *p < 0.001

### Preferred approaches or measures towards securing primary care

Table 4 provides an overview of the approaches and concepts that could make a greater or lesser contribution to securing primary care from the respondents’ point of view (see Table 4). The results clearly showed respondents to favour measures towards enhancing the position of general practitioners in the healthcare system and adapting continuing medical education to current requirements. Respondents also showed a striking level of support for a primary care system as a measure towards a sustainable increase in the ability of primary care to function and therefore its attractiveness, potentially in combination with a more authoritative primary care service catalogue.

**Table 4:** Agreement with measures towards securing primary care

		Rotated component matrix			
**Question:** *Selected measures are listed below. How effective you think each measure would be in securing primary care in the long term?* (N = 5,164; response categories *very effective* and *somewhat effective* taken together)	Overall agreement	Comp. 1 (variance clarif.: 39.7%)	Comp. 2 (variance clarif.: 15.6%)	Comp. 3 (variance clarif.: 11.6%)	Urban vs. rural physicians
A primary care system with general practitioners as the first point of contact for patients while avoiding simultaneous appointments with specialists without prior referral	88%	.521	.069	.775	84%/92%
Routine establishment of complementary longitudinal programmes alongside Medicine courses communicating interest, insights, and skills needed in general practice	78%	-.006	.488	.54	79%/77%
Substantial reduction in general cost pressure for general practitioners	69%	.65	.45	.134	66%/72%
Shift away from classical medical practices towards outpatient (primary) care centres with the aim of expanding primary care; examples include polyclinics or health centres near hospitals or in urban areas towards fostering multi-professional cooperation and other more flexible working models	62%	.866	.144	.026	64%/60%
Substantial increase in the proportion of primary care in continuing medical education (such as an increase to a third)	59%	.411	-.009	.402	57%/61%
An authoritative primary care service catalogue as a clear guide to what can be expected from a GP towards preventing primary care overload, such as by ensuring sufficient qualifications and a set number of working hours	58%	.715	.485	.040	55%/61%
Fundamental general medical training reforms (including shortening and flexibilisation, more focus on key competencies in primary care)	55%	.793	-.035	.239	55%/55%
Medical study course and curriculum restructuring (improvement with more specific and relevant preparation for a future career in outpatient clinics and medical practices)	54%	.31	.401	.753	58%/51%
Major changes to the enrolment criteria for medical study courses (broader and more intensive inclusion of factors such as personality and curriculum details)	51%	.159	.797	.359	51%/51%
Fundamental improvement in general practitioner pay (such as pegging it to at least specialist level)	49%	.101	.618	.223	45%/52%
Delegation and increased use of non-medical health professions and extension to their sphere of responsibility	45%	.869	.029	.163	31%/58%*
A substantial increase in study places for Human Medicine	42%	.827	.007	.281	33%/50%*
Effective recruitment of medical personnel (more effort on incentives and rewards such as in municipalities with subsidies and bonuses for establishment in a rural area, for example)	39%	.852	.155	.074	32%/46%*
Consistent establishment of a rural primary care quota across Germany (clearly regulated in each federal state, with on-top quotas as required)	36%	.282	.674	-.339	24%/48%*
(More effective) demand planning with distribution aimed towards regional effectiveness	34%	.84	.199	.081	34%/34%
Increased and more standardised use of digitalisation and telemedicine (including video consultations as well as health app prescriptions for patient self-management)	29%	.368	.079	.403	41%/17%*
Quotas for access to specialist training	25%	.326	.762	-.352	25%/25%
Provide career changers from other disciplinary backgrounds with more access and authorisation to work as a general practitioners	22%	.777	.252	-.388	23%/21%

Extraction method: Principal component analysis

Rotation method: Varimax, Kaiser normalisation

Rotation convergence in six iterations

Total variance clarified: 66.9%; sampling suitability according to Kaiser–Meyer–Olkin:.653

Significance according to Bartlett: *p* < 0.001

Commonalities in all included variables above limit.5

Difference significant at *p < 0.001

*“General practitioners need reinforcement in their decision-making authority. In the current situation, this would require a primary care system that can live up to its name.”* (Open response from a responding GP [m])

Beyond changes to medical study courses (enrolment criteria, restructuring subject matter), respondents were in favour of structurally sound interventions during study courses (longitudinal support programmes) for students to gain more in-depth insights into the activities of general practitioners, gain interest and motivation, dispel reservations, and to learn the skills needed early on in the course. Many general practitioners were also convinced of the importance of continuing medical education and (increased) development of multi-professional care centres to counteract the fragility of a nationwide primary care system and put synergy effects to more effective use in interdisciplinary networking. Many of the respondents also saw outpatient primary care centres as an important instrument in meeting the employment expectations of young physicians more effectively.

*“Polyclinics do indeed promote a shift away from traditional practices; that could be seen as a positive and a negative. Even so, this is a great opportunity to free general practitioners from their existence as lone warriors, modernise the field, and create stronger connections with other professional groups.”* (Open answer from a responding GP [m])

*“It makes sense in principle – the physicians there work on their own account, but they also share some of the costs; teamwork is very important. […] My own daughter works in a medical centre and wouldn’t dream of switching to a conventional medical practice anymore.”* (Open answer from a responding GP [f])

The physicians surveyed showed relatively low confidence in measures such as rural GP quotas, budget increases, or digitalisation measures towards stabilising primary care in the long term. Most respondents also did not see increasing the number of study places to be sufficient on its own.

*“I am far from convinced that more study places would automatically lead to more doctors. The system is fundamentally designed to focus on producing specialists. That’s exactly what needs to be changed.”* (Open answer from a responding GP [m])

Factor analysis combining variables into factors based on underlying relationships as correlations [43] revealed three distinct clusters of general practitioners that would each recommend varying measures in combination. The largest cluster clearly favoured a structural improvement in the role of general practitioners and opportunities for action in the healthcare system (including primary care, relief from cost pressure, new practice models, primary care service catalogue, delegation of activities). In contrast, the other two clusters emphasised changes in study courses and continuing medical education towards stabilising and improving the influx of young medical students, but also to prepare them more effectively for later work in primary care (including longitudinal support programmes, restructuring curricula and subject matter covered in medical study courses, and changes in enrolment criteria for medical study courses).

Urban physicians showed a highly significant difference in the measures they favoured compared to their rural colleagues. Urban physicians were far more in favour of primary care centres, and also saw substantially greater opportunities in digital solutions. In contrast, physicians from rural practice locations saw greater added value in the increased use of delegation and non-medical health professions; they were also more in favour of effective medical recruitment for physicians to establish practices in rural areas, and were considerably more open to a nationwide, uniformly regulated quota for rural physicians compared to their urban colleagues.

*“We need to get even further away from the idea that everything depends on general practitioners. There’s still a lot of potential here. This will take upgrading non-medical professions to enable delegation. This principle works much better in other countries than it does here.”* (Open answer from a responding GP [f])

*“These problems will get worse if we don’t intervene more heavily in the entire distribution system. The deciding factor needs to be the data available on how great the need is for different disciplines. This would mean general medicine receiving a larger fixed quota in continuous medical training, possibly with the freedom to choose a profession giving way to requirement.”* (Open answer from a responding GP [m])

Another noticeable result was that far more physicians with current or previous academic teaching experience in primary care advocated for methodical establishment of supplementary longitudinal accompanying programmes alongside medical study courses than did other physicians (93% versus 68%, *p* < 0.001). The same applies to restructuring the subject matter and curricula in medical study courses as well as major changes in enrolment criteria for medical students.

*“I’ve seen it from my own experience as a teaching doctor: These accompanying programmes open up new horizons; they provide close guidance and motivation. […] They expose students to the broad-based activities of general practitioners in practice. […] General practitioners could be involved in this process to great benefit. I see these models guiding us into the future.”* (Open answer from a responding GP [f])

### Assessment of measures taken towards securing primary care as well as further optimisation approaches to be adopted

The general conclusion from most respondents on their general impression of measures taken so far towards securing primary care generally ranged between reserved and sceptical. Over a third of respondents at 37% were very (5%) or somewhat satisfied (32%) with health policy efforts taken so far towards stabilising primary care, leaving 63% who were either very (19%) or somewhat dissatisfied (44%). The open questions repeatedly echoed the recent increase in the number of specialist physicians being licensed, but this was still far too low to cover the foreseeable demand for new general practitioners. Many respondents therefore took an ambivalent view of the measures taken so far as expressed in these sample free-text answers:

*"The efforts taken in the last few years are fundamentally right, but they still lack enough impetus and motivation considering the extent of the shortage of physicians we will be facing in the next few years. [...] There is a real danger that we will drop the ball on this development, trying to play catch-up ever after.”* (Open answer from a responding GP [m])

*“There’s a lot of experimentation going on at the moment, but it still leaves the problem of primary care not being attractive enough for new people to join.”* (Open answer from a responding GP [f])

Respondents’ criticism focused on various specific areas alongside the limited resources allocated by healthcare policymakers towards supporting primary care. The first of these areas involved medical study course curricula and subject matter, which respondents thought needed to be oriented far more towards outpatient care. According to a number of respondents, the Masterplan 2020 reform for medical study courses initiated in Germany has been half-hearted in its curricular readjustments, which have not yet been implemented throughout.

*“It still hasn’t happened. […] Students need far more thorough exposure to primary care. I don’t mean quotas for the most part, but insights, motivation, and skills. We still have a major shortfall in this respect.”* (Open answer from a responding GP [m])

*“Study materials need to increase their focus on primary care in particular and the outpatient sector in general, and everything that goes with it.”* (Open answer from a responding GB [f])

A striking number of respondents were hesitant about rural primary care quotas, seeing little positive effect in the current design for a substantial increase in the number of upcoming general practitioners. This was largely attributed to many federal states not deciding on additional quotas for the rural primary care quota that has been partly established but earmarking a proportion of the existing study places for the quota instead. There was also criticism of what was seen as the inconsistent planning in the rural primary care quota within Germany’s federal structure. Many general practitioners saw more benefit in increasing efforts towards finding and selecting future general practitioners.

*“We’re well acquainted with the quota discussions and solutions by now from other areas. Quotas rarely provide an effective solution; instead, they reinforce the image that someone needs additional help due to the dire situation they’re in.”* (Open answer from a responding GP [f])

*“Upcoming general practitioners are a different kind of beast. We need to look more closely at this to attract the right people and motivate them at an early stage. […] They don’t have to be the best and the brightest the schools have to offer in terms of grades; general practitioners in particular are people-oriented types with life experience. […] The system needs to reward personality and previous experience even more.”* (Open answer from a responding GP [f])

Reform efforts can be seen in medical studies, but the respondents still saw too much inertia with a corresponding backlog of reforms in continuing medical education.

*“There are problems with continuing medical education in various areas. It isn’t compact enough, and it’s not close enough to the reality of primary care either. Skills aren’t taught to prepare for real life in primary care, and there is also too little current knowledge such as evidence-based medicine and the opportunities of digitalisation in healthcare.”* (Open answer from a responding GP [m])

Some of the respondents saw competence centres for primary care that have been established in almost all federal states as they actively support continuing medical education – a valuable instrument in promoting general practitioner recruitment from continuing medical education.

*“The idea that universities should maintain their prime role in training medical professionals is highly important and contributes to professionalization.”* (Open answer from a responding GP [m])

*"These institutions could make a decisive contribution towards improving preparation and enthusiasm for the job while also to reducing dropout rates in continuous medical training. [...] They need to be given even more support and a more meaningful role."* (Open answer from a responding GP [f])

Many of the respondents also expressed hopes for a reduction in the constant pressure and influence of cost restrictions that general practitioners are exposed to together with an increase in their independence and sovereignty in the healthcare system. Some of the responding physicians again emphasised the importance of establishing a primary care system as a structured model based on general practitioners. The term *primary care system* is often used as a code for securing greater capacity and sovereignty for general practice, which cannot develop its full potential due to the structural constraints in the current healthcare system according to many respondents. However, the result is a professional field that is less attractive for newcomers.

*"I’d like to see us GPs no longer being tied to the leash of bureaucracy and cost guidelines. [...] It’s extremely unsettling dealing with the constant threat of recourse. [...] I’d also like to see better healthcare management where we’re no longer the ones who have to clean up the mess from misalignments that we had no hand in. [...] A different public image of us would also emerge as a result."* (Open answer from a responding GP [m])

Most of the general practitioners consulted (65%) expressed a desire for their professional representatives to be more involved in health policy-relevant committees for them to bring their views to the table and help shape measures towards counteracting combating a potential shortage of general practitioners in the future. The following question gave respondents an opportunity to choose between three approaches that would help integrate views from GPs more effectively in the healthcare system. Under a third at 30% spoke in favour of increasing the number of general practitioners in medical and scientific committees focused on health policy, while 25% spoke for stronger and more systematic approach to federal, state, and local health policy protagonists from organised associations and professional societies such as general practitioners’ associations and the German Society of General Practice and Family Medicine (DEGAM). Nearly half at 43% were in favour of a proposal put forward in 2023 by the German Medical Association (BÄK) calling for the establishment of a cross-departmental German Health Council involving the German Medical Association and other representatives from self-government and research. Like the German Ethics Council, the German Health Council would participate in political processes proactively or on behalf of the respective specialist department.

Despite current concerns on stabilising general outpatient care, respondents considered a career in primary care to be more important than ever, especially in times of increasing disciplinary specialisation. Effective measures towards reinforcing and revitalising the general practitioner profession would safeguard the healthcare system as a whole.

*“There is a great need for competent all-rounders in a healthcare system becoming increasingly fragmented and specialised. I don’t see any alternative to bringing primary care into a new era.”* (Open answer from a responding GP [f])

*"It would take a wide range of adjustments and tweaks towards fully modernising, relieving, and upgrading primary care for us to return to what we once were: the backbone of healthcare. [...] But there’s a lot to do."* (Open answer from a responding GP [m])

## Discussion

### Main findings

Primary care will play a key role in an efficient and resilient healthcare system in the future [1, 3, 24, 45]. The problem of declining primary care has reached the core of the general practitioner profession as the results from the present survey on 5,164 general practitioners confirm. A high percentage of those surveyed reported experiencing stress and bottlenecks due to a healthcare system noticeably thinning out in some regions. This applies not only to the decline in general practices, but also specialist medical practices and especially in small towns and rural communities.

The results from the present contribution show that many general practitioners are concerned about the future in securing long-term primary care. Despite some progress in strengthening primary care, there is a great need for action in the coming years to increase the profession’s attractiveness for young medical professionals and keep the number of general practitioners at a stable level. The respondents saw the greatest issues in structural problems in the German healthcare system as well as deficiencies in foundational and continuing medical education. Deficits especially in regional demand planning and incentive support add to the problem.

**Fig. 1:**
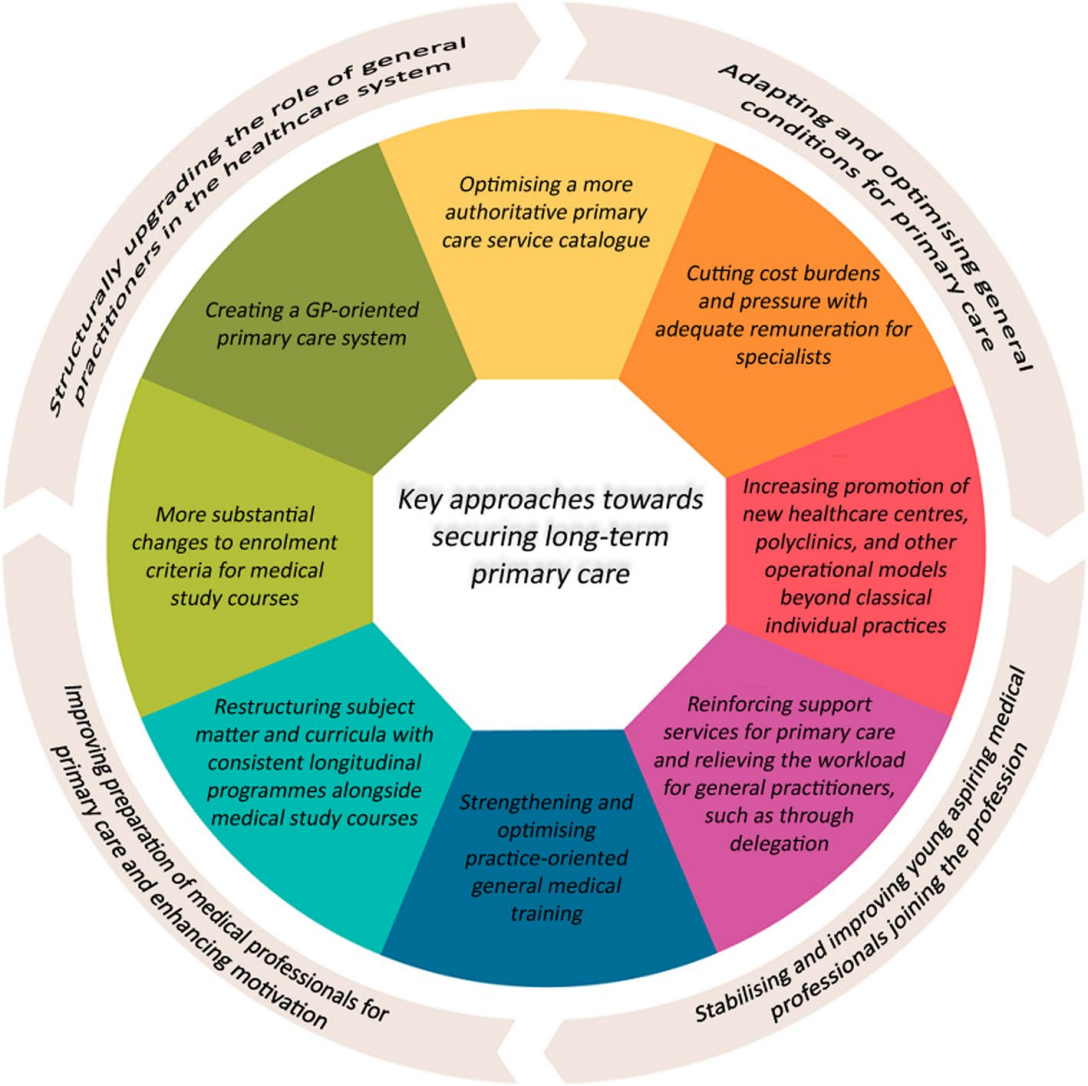
Concentrating the main findings into major dimensions of optimisation for primary care

The present study findings demonstrate that the general practitioners surveyed have set clear priorities on expectations for long-term stabilisation towards safeguarding primary care. Figure 1 summarises the key demands. These coincide with other studies and conclusions from expert opinions in many respects as we will be discussing below (see Fig. 1).

### Comparison with previous studies

A number of stakeholders in healthcare have issued methodical reviews emphasising the necessity of available and adequately equipped general practices. These are seen as essential for securing effective primary care and for coordinating treatment pathways and specialist care. In this way, they contribute significantly to the quality and efficiency of the healthcare supply chain [45]. International studies on controlling access to needs-based specialist care (general practitioner gatekeeping models) have demonstrated this. Clear signs of an increasing fragility in the healthcare system have been appearing in contradiction to the above tenets on the vital role of general medicine.

Findings on the experience of dwindling healthcare according to the health report prepared by MLP SE in 2022, a representative survey of physicians commissioned by the German demoscopic institute (IfD) with repeated focus on outpatient care, largely tally with the overall perceptions and attitudes found in the present study [41]. Comparing the trend in previous MLP health reports demonstrates a sharp rise in the percentage of physicians from 31% in 2016 to 52% perceiving a substantial shortage of practices in their own region. This percentage is far above average at 76% in structurally weaker regions.

The measures favoured by general practitioners as broadly surveyed in the present contribution in a large sample reveal a high level of agreement with a previous qualitative interview study on general practitioners [38]. Similarities are immediately apparent between general practitioners and proposals from experts in healthcare services research and the German healthcare advisory committee (SVR) [46]. This is particularly true with regard to certain approaches aimed at securing general medical care. Beyond a reduction in cost pressure and restrictions [40, 47], most respondents see a primary care physician system as an effective strategy [30]. The German Society of General Practice and Family Medicine (DEGAM) and other general medical organisations has been calling for such a system for years to reposition primary care in the healthcare system and create more favourable conditions for its availability [29].

The SVR report and other expert opinions have also called for increased steps towards GP-oriented care [45]. This is justified not only in the increased attractiveness of primary care medicine, but also from existing evidence on its added value including lower five-year mortality, lower risk of hospitalisation, and increased continuity of care [48–51]. Achieving greater focus on general practitioners will entail further optimisation in primary care regarding quality and quantity following international models, according to the committee. However, this will require a substantial increase in appropriately qualified specialists in general medicine.

Further overlaps can be identified between the general practitioners surveyed and the demands made by experts. These relate to the concretisation of a primary care service catalogue as an authoritative reference wherever possible. Another shared priority is the establishment and continuous promotion of multi-professional care centres, which present an opportunity for synergies through multi-disciplinary solutions [19, 52]. The same applies to strategies towards extending delegation options, especially calling on non-medical health professions [31, 53], as well as stronger regulation in accessing specialist training, which should be more closely matched to general demand [30, 36].

The present survey results also demonstrate the importance of obtaining views from general practitioners on the topic as they set their own priorities and focuses, which may sometimes differ from the expertise available at the time. This is especially evident in longitudinal programmes alongside study courses, which respondents consider to be highly effective in raising interest in primary care amongst students at an early stage as well as imparting knowledge and skills for a career in primary care. The Masterplan 2020 project adopted in Germany a few years ago specifically included longitudinal programmes held by general practitioners as a core element [54]. The SVR report also highlighted the importance of additional options in general medical study courses to include voluntary career paths for physicians in rural areas or increased career research [46]. International studies have shown participation in general medicine block placements to have a substantial impact on career preference and willingness to open a medical practice [40, 55, 56]. Medical faculties at universities should therefore focus more on communicating knowledge and experience especially regarding medical careers in rural settings to students [27, 32, 56]. The authors noticed in researching the present contribution that other authors in German-speaking countries, such as van den Bussche et al. [30, 36, 57], do not give longitudinal programmes as much prominence as they do to other demands for primary care reform.

Many general practitioners see a great need to modernise traditional work and employment customs as modern models oriented towards the next generation, while also substantially improving the image of primary care [58]. Poignantly, a relatively large group spoke in favour of new, integrative settings such as primary care centres leading away from classical individual practices [59–61]. This is also partly reflected in studies by Huenges et al. [62], Roick et al. [59], van den Bussche et al. [30, 37, 57], and other authors who surveyed physicians in training and advanced medical students [63–65]. These studies revealed the need for a more far-reaching reform of continuing medical education in terms of duration, subject matter, and teaching method. Competence centres such as those that the SVR has suggested as nationwide, university-affiliated institutions [46] could provide effective support as obvious partners in connection with study courses. These competence centres could also provide support for continuous medical education and help strengthen the next generation of general practitioners with structured course programmes and mentoring or establishing additional training networks [36]. Finally, they could help reduce dropout rates in continuing medical education by improving preparation and conveying realistic expectations [66]. Studies such as those by Linde et al. have shown a strong orientation towards disease and serious illness in medical study courses and clinical training, while largely ignoring aspects that shape healthcare given in independent practices in general and primary care in particular, especially regarding the consequences of working in areas of low prevalence. According to several studies, new physicians often find the transition difficult, experiencing a real “practice shock” from a massive divergence in working as a physician from studies and continuous medical training, turning their career entry into a jarring experience [40, 56, 59, 62].

General practitioners noticeably saw some high-profile approaches discussed in the health policy discourse as only being of limited help. Rural GP quotas are one such example; many respondents did not see much benefit here. Criticism has been levelled at the regional nature of the scheme being limited to federal states, and the very limited capacities by numbers in the rural GP quota model adopted so far. In addition, the preliminary study revealed the apprehension amongst general practitioners that the quota principle could emphasise the deficient state of primary care in the public eye; this itself could diminish the attractiveness of the profession for young physicians, rendering the measure unsustainable [38, 67, 68]. In contrast to the expert opinions presented, our respondents took a more reserved position on allowing more career changers to enter general practice and lowering barriers to continuing medical education [29, 30, 57, 69]. Concerns about lowering the quality standards indispensable to general practitioners were also reflected in DEGAM positions.

Respondents expressed a desire for general practitioners to be involved in planning, implementing, and evaluating measures consistently towards counteracting the impending shortage of primary care at municipal and federal state level, and in federal healthcare policy. Strategies and best practice examples already exist to this end [32, 70–73]. General practitioners are usually represented as a minority with their association in medical and research committees, warranting greater attention from policymakers in healthcare. Closer cooperation between the professional society and health policies in the states and municipalities would also be conceivable.

### Strengths and limitations

The survey developed from a qualitative preliminary study and was therefore tailored to the perspective of primary care in a specific real-life approach. We were able to collect non-standardised responses from a series of open questions. The survey achieved a relatively high response rate, allowing us to diversify the sample in broad terms of characteristics and attitudes towards the study topic. The reasons for the exceptionally high response rate may be, on the one hand, that general practitioners perceive the topic as extremely important and urgent. This can be interpreted as a reference to the lack of larger studies that give general practitioners the opportunity to contribute their own viewpoints on ensuring primary care. Although the questionnaire used was detailed, the use of open-ended questions gave respondents the chance to express their opinion and put forward a variety of arguments. At the same time, the authors already have experience in conducting physician surveys in the relevant federal states. Experience has shown that the combination of a postal invitation letter and an online survey has proven particularly promising.

Even so, there are limitations. The study cannot claim to be representative in the strict sense due to the limited number of cases and regional recruitment strategy, at most approximating to a representative study due to comparison with health insurance data. It is also possible that physicians with an interest in the topic participated more closely in the study. The fact that the study is limited to the situation in the German healthcare system creates further limitations in the ability to easily transfer the results to other healthcare systems.

We would also like to note that these are the viewpoints and opinions of the general practitioners surveyed on securing effective primary care. The authors consider the present work to add to studies on subjects such as career trajectories and departures amongst prospective physicians during their studies and in continuing medical education. This survey only included physicians already active in healthcare; this gives them a specific perspective on the topic at hand.

The need to create a compact survey led to prioritisation in designing the survey instrument. A more intensive survey with questions on personal motivation in choosing a particular career or specialty or the advantages of working as a general practitioner would have been interesting but was not feasible. The same applies to a questionnaire on individual measures and on how individual general practitioners could contribute to raising the attractiveness of primary care as a career option. These would be topics that a follow-up study could focus on in more detail.

Last but not least, there are limitations with regard to statistical evaluations. For example, a certain limitation is the lack of multivariate explaining analysis, which, however, was not intended due to the exploratory nature of the study.

## Conclusions

Securing primary care poses a complex, multifactorial set of challenges for policymakers in national healthcare. General practitioners may have seen some progress in recent years, but still see a substantial need for action to prevent a major decline in the number of general practitioners. This is closely linked with a worsening local healthcare situation, which many respondents have already observed. The issue involves a comprehensive attractiveness problem leading to an insufficient induction of new physicians into general practice according to the opinion of the correspondents. The general practitioners responding to the survey in the present contribution suggested that establishing a primary care system or enhanced role for general practitioners would be an effective approach to reinforcing primary care. In addition, respondents expressed the benefit of promoting greater interest and contact with general practice in training and foundational and continuing medical education while also restructuring curricula and enrolment criteria for medical study courses and reforming and reinforcing foundational and continuing medical education in general medicine. Finally, many respondents saw a stronger transition from classical general practices to multi-professional healthcare centres as a valuable contribution.

## Supplementary Information

Supplementary Material 1. Questionnaire.

## Data Availability

All major data generated or analysed during this study are included in this published article. Additional information can be provided on request made to the corresponding author.

## Abbreviation

GP(s): General Practitioner(s)
