# Supplementary material for: Approaches towards averting a potential structural shortage of general practitioners: results of a quantitative survey on attitudes, experiences, and ideas from general practitioners in the Federal Republic of Germany
Source: BMC Prim Care. 2025 Apr 24;26:124. doi: 10.1186/s12875-025-02840-y (PMC12023567; doi:10.1186/s12875-025-02840-y)
Supplement: Supplementary file 1 — Supplementary Material 1. Questionnaire. [file 12875_2025_2840_MOESM1_ESM.pdf]

1. In your opinion, will healthcare in Germany improve or worsen overall in the coming years?

◯ Substantially improve ◯ Somewhat improve ◯ Remain the same ◯ Somewhat worsen ◯ Substantially worsen

◯ Difficult to say, don’t know

2. In your opinion, how would you rate this for outpatient care in particular, that is, for general practitioners and specialists? Do you think these areas of healthcare will…

◯ Substantially improve ◯ Somewhat improve ◯ Remain the same ◯ Somewhat worsen ◯ Substantially worsen

◯ Difficult to say, don’t know

3. Have you been seeing any increase or decrease in the attractiveness of primary care for young physicians compared to the situation a few years ago?

◯ Substantial increase ◯ Some increase ◯ Some decrease ◯ Substantial decrease

◯ Difficult to say, don’t know

4. What do you think has led to this? You may answer in keywords and bullet points.

____

____

____

5. Would you currently recommend medical students or trainee physicians to aim for a profession as a general practitioner, or would you tend towards advising against it?

◯ Definitely recommend it ◯ Somewhat recommend it ◯ Somewhat advise against it ◯ Definitely advise against it

◯ Difficult to say, don’t know

6. Why do you take this view? You may answer in keywords and bullet points.

____

____

____

7. In your experience, how much of an impact have the following issues had on your activities as a general practitioner?

	Extreme	Somewhat	Not so much or not at all	Difficult to say
Bureaucracy (such as reporting and documentation obligations)	○	○	○	○
Difficulties in finding staff (such as practice staff, physicians for employment)	○	○	○	○
Cost pressure and restrictions in healthcare (such as those hindering optimised care for each patient as an individual)	○	○	○	○
Additional burdens from the shortage of physicians (such having to provide more patient care at your own practice due to other general practices closing down for lack of succession)	○	○	○	○
Lack of specialists available in your area for you to perform your own obligations in guiding your patients through the system properly	○	○	○	○
Other; please elaborate:	○	○	○	○

8. Thinking about the future and development opportunities in primary care over the next ten to twenty years, would you say you were more confident or worried?

○ Very confident ○ Somewhat confident ○ Somewhat worried ○ Very worried

○ Difficult to say, don't know

9. What major changes do you see in the future for primary care? You may answer in keywords and bullet points.

____

____

____

10. Thinking about the long term, how secure would you say that primary care in Germany will be in the coming decades?

◯ Very secure ◯ Somewhat secure ◯ Somewhat insecure ◯ Not at all secure

◯ Difficult to say, don't know

11. In your opinion, what are the greatest challenges and issues facing primary care in the long term? You may answer in keywords and bullet points.

____

____

____

12. a) Do you own your own medical practice?

◯ Yes ◯ No – go straight to **Question 13**

12. b) Imagine you opted to discontinue your medical practice. How easy or difficult would you expect it to be to find a successor?

◯ Very difficult ◯ Somewhat difficult ◯ Somewhat easy ◯ Very easy

◯ Difficult to say, don't know

13. a) In your opinion, how severely has the decline in primary care and general practices impacted the region around your practice?

◯ Very heavily impacted ◯ Somewhat impacted ◯ Somewhat affected ◯ Not impacted much or at all => go straight to **Question 14** ◯ Difficult to say, don't know

13. b) Based on the decline in primary care that you have confirmed, have you already noticed signs of a healthcare shortage in your area (such as problems that remaining practices might be having taking on additional patients), or currently not so?

◯ Clear signs of shortage ◯ Some signs of shortage ◯ No signs of shortage yet ◯ Difficult to say, don't know

14. Imagine the primary care situation in ten to twenty years. Do you see a serious widespread shortage of general practitioners throughout Germany, or do you see a shortage mainly in rural and structurally weak regions, or do you not see any significant shortage of general practitioners? (Only one answer, please.)

◯ Significant, widespread shortage of general practitioners

◯ (Increased) shortage of general practitioners in rural and structurally weak regions

◯ No significant shortage of general practitioners => go straight to **Question 16**

◯ Difficult to say, don't know

15. Thinking about the general healthcare demand, how severe do you think the shortfall in general practitioners will be in ten to twenty years? A rough ballpark percentage will do.

◯ 5 to 10 percent ◯ 10 to 15 percent ◯ 15 to 20 percent ◯ More than 20 percent

◯ Difficult to say, don't know

16. In your opinion, what conditions would seem especially favourable and what measures should be prioritised towards securing primary care in the long term? You may answer in keywords and bullet points.

____

____

____

17. Selected measures are listed below. How effective you think each measure would be in securing primary care in the long term?

	Very effective	Somewhat effective	Not or not very effective	Difficult to say
A substantial increase in study places for Human Medicine	◯	◯	◯	◯
Major changes to the enrolment criteria for medical study courses (broader and more intensive inclusion of factors such as personality and curriculum details)	◯	◯	◯	◯
Medical study course and curriculum restructuring (improvement with more specific and relevant preparation for a future career in outpatient clinics and medical practices)	◯	◯	◯	◯

Routine establishment of complementary longitudinal programmes alongside Medicine courses communicating interest, insights, and skills needed in general practice	○	○	○	○
Consistent establishment of a rural primary care quota across Germany (clearly regulated in each federal state, with on-top quotas as required)	○	○	○	○
Fundamental general medical training reforms (including shortening and flexibilisation, more focus on key competencies in primary care)	○	○	○	○
Effective recruitment of medical personnel (more effort on incentives and rewards such as in municipalities with subsidies and bonuses for establishment in a rural area, for example)	○	○	○	○
(More effective) demand planning with distribution aimed towards regional effectiveness	○	○	○	○
Substantial increase in the proportion of primary care in continuing medical education (such as an increase to a third)	○	○	○	○
Quotas for access to specialist training	○	○	○	○
Provide career changers from other disciplinary backgrounds with more access and authorisation to work as a general practitioner	○	○	○	○
An authoritative primary care service catalogue as a clear guide to what can be expected from a GP towards preventing primary care overload, such as by ensuring sufficient qualifications and a set number of working hours	○	○	○	○
Substantial reduction in general cost pressure for general practitioners	○	○	○	○
Fundamental improvement in general practitioner pay (such as pegging it to at least specialist level)	○	○	○	○
Introduction of a primary care system with general practitioners as the first point of contact for patients while avoiding simultaneous appointments with specialists without prior referral	○	○	○	○
Shift away from classical medical practices towards outpatient (primary) care centres with the aim of expanding primary care; examples include polyclinics or health centres near hospitals or in urban areas towards fostering multi-professional cooperation and other more flexible working models	○	○	○	○
Delegation and increased use of non-medical health professions and extension to their sphere of responsibility	○	○	○	○

Increased and more standardised use of digitalisation and telemedicine (including video consultations as well as health app prescriptions for patient self-management)	◯	◯	◯	◯
Other; please elaborate:	◯	◯	◯	◯

18. If there were <u>one</u> measure alone that would secure primary care in the long term, which one would you consider to be the most beneficial or especially urgent?

____

19. Recent years have seen various efforts in health policy towards supporting and securing primary care. How satisfied are you with the overall measures taken so far? (Try to summarise your general impression or observations on this issue.)

◯ Very satisfied ◯ Somewhat satisfied ◯ Somewhat dissatisfied ◯ Very dissatisfied

◯ Difficult to say, don't know

20. What makes you satisfied or dissatisfied, what major factors do you see at play here?

____

____

____

21. Where do you see a particular need for changes or reinforcement in efforts towards ensuring primary care in the long term? You may answer in keywords and bullet points.

____

____

____

22. Thinking about measures against a potential shortage of general practitioners in the future, do you see general practitioners as being sufficiently represented in health policy-relevant committees enabling them to share their opinions and help shape measures, or is there still some catching up to do in this regard?

◯ Sufficient representation ◯ Some catching up needed ◯ Great need to catch up

◯ Difficult to say, don't know

23. What would improve integrating the opinions and views from primary care when deciding on measures to counteract a shortage of general practitioners? Select the point that seems most promising to you. (Only one answer, please.)

◯ Increasing the number of GPs in medical and research committees focusing on health policy or those involving policymakers in healthcare

◯ More methodical and efficient approach in addressing health policy protagonists at federal, state, and local level by organised associations and professional societies such as the German Society of General Practice and Family Medicine (DEGAM)

◯ Proposal from the German Medical Assembly (2023): Establishment of a cross-departmental German health council involving the German Medical Association (BÄK) and other representatives from self-government and research. This would also include general practitioners. Like the German Ethics Council, the German Health Council would participate in political processes proactively or on behalf of the respective specialist department.

◯ Other:

We would like to ask you for some information for statistical purposes. As with the rest of the questionnaire, the information you give will of course be treated in strict confidence and anonymity.

Are you…

◯ Male ◯ Female ◯ Diverse

Your **age**: ______

Where is your medical practice located? In a municipality or city with a population of…

◯ more than 100,000 ◯ 20,000 to 100,000 ◯ 5,000 to 20,000 ◯ less than 5,000 inhabitants

Which of the following most accurately describes your **medical practice**?

◯ Individual practice (you are the only physician) ◯ Individual practice with employed physicians on staff

◯ Group practice ◯ Medical care centre or polyclinic ◯ Other

How many patients come to your practice for treatment each quarter?

◯ 500 to 750 ◯ 751 to 1,000 ◯ 1,001 to 1,500 ◯ 1,501 to 2,000 ◯ More than 2,000

Have you worked or are you working as **an academic teaching physician**; that is, have you already been involved in training medical students such as in trainee or clinical internships or general medicine block placements, or held medical lectures or seminars?

◯ Yes, currently working as a teaching physician ◯ Yes, previously working as a teaching physician ◯ No

<u>Thank you for your time!</u>

Is there anything else you would like to tell us? Use this space for suggestions, comments, and criticisms.

____

____

____
